# Distinct transcriptome responses to water limitation in isohydric and anisohydric grapevine cultivars

**DOI:** 10.1186/s12864-016-3136-x

**Published:** 2016-10-20

**Authors:** Silvia Dal Santo, Alberto Palliotti, Sara Zenoni, Giovanni Battista Tornielli, Marianna Fasoli, Paola Paci, Sergio Tombesi, Tommaso Frioni, Oriana Silvestroni, Andrea Bellincontro, Claudio d’Onofrio, Fabiola Matarese, Matteo Gatti, Stefano Poni, Mario Pezzotti

**Affiliations:** 1 Dipartimento di Biotecnologie, Università di Verona, 37134 Verona, Italy; 2 Dipartimento di Scienze Agrarie, Alimentari e Ambientali, Università di Perugia, 06128 Perugia, Italy; 3 E. & J. Gallo Winery, Modesto, CA 95353 USA; 4 Istituto di analisi dei sistemi ed informatica “Antonio Ruberti”, Consiglio Nazionale delle Ricerche, Roma, Italy; 5 Dipartimento di Scienze Agrarie, Alimentari ed Ambientali, Università Politecnica delle Marche, 60131 Ancona, Italy; 6 Dipartimento per l’Innovazione dei Sistemi Biologici, Agroalimentari e Forestali, Università della Tuscia, Viterbo, Italy; 7 Dipartimento di Scienze Agrarie, Alimentari ed Agro-Ambientali, Università di Pisa, 56124 Pisa, Italy; 8 Dipartimento di Scienze delle Produzioni Vegetali Sostenibili, Università Cattolica del Sacro Cuore, 29122 Piacenza, Italy

**Keywords:** Transcriptome, Grapevine, Photosynthesis, Water stress and stomatal behavior

## Abstract

**Background:**

Grapevine (*Vitis vinifera* L.) is an economically important crop with a wide geographical distribution, reflecting its ability to grow successfully in a range of climates. However, many vineyards are located in regions with seasonal drought, and these are often predicted to be global climate change hotspots. Climate change affects the entire physiology of grapevine, with strong effects on yield, wine quality and typicity, making it difficult to produce berries of optimal enological quality and consistent stability over the forthcoming decades.

**Results:**

Here we investigated the reactions of two grapevine cultivars to water stress, the isohydric variety Montepulciano and the anisohydric variety Sangiovese, by examining physiological and molecular perturbations in the leaf and berry. A multidisciplinary approach was used to characterize the distinct stomatal behavior of the two cultivars and its impact on leaf and berry gene expression. Positive associations were found among the photosynthetic, physiological and transcriptional modifications, and candidate genes encoding master regulators of the water stress response were identified using an integrated approach based on the analysis of topological co-expression network properties. In particular, the genome-wide transcriptional study indicated that the isohydric behavior relies upon the following responses: i) faster transcriptome response after stress imposition; ii) faster abscisic acid-related gene modulation; iii) more rapid expression of heat shock protein (HSP) genes and iv) reversion of gene-expression profile at rewatering. Conversely, that reactive oxygen species (ROS)-scavenging enzymes, molecular chaperones and abiotic stress-related genes were induced earlier and more strongly in the anisohydric cultivar.

**Conclusions:**

Overall, the present work found original evidence of a molecular basis for the proposed classification between isohydric and anisohydric grapevine genotypes.

**Electronic supplementary material:**

The online version of this article (doi:10.1186/s12864-016-3136-x) contains supplementary material, which is available to authorized users.

## Background

Grapevine (*Vitis vinifera* L.) is one of the oldest and most significant horticultural crops in the world, not only for its economic and social impact, but also for its strong and ancient connection with human culture and civilization [1]. Many geographical areas traditionally devoted to viticulture are also severely affected by drought and freshwater limitations due to global climate change and the need for more irrigation [2]. However, the diversity of grapevine genetic resources encompasses ~10,000 different varieties [3] which differ substantially in terms of drought tolerance [4].

Grapevine is categorized as “drought avoiding” [5] or “pessimistic” according to the ecological classification introduced by Jones [6] where “pessimists” are genotypes that adapt to preserve their water status under drought conditions and utilize future resources more conservatively, whereas “optimists” have looser stomatal control and do not conserve water. The physiological classification proposed by Stocker [7] and by Tardieu and Simonneau [8] defines plants as isohydric if they can maintain a constant midday leaf water potential (Ψ_leaf_) regardless of soil water availability, or anisohydric if Ψ_leaf_ significantly declines with evaporative demand during the day, and is typically lower in water stressed (WS) compared to well-watered (WW) plants. Grapevine cultivars of different geographical origins can fall within one or other of these categories [9]. Drought tolerance in grapevine is primarily mediated by changes in stomatal conductance [10]. Based on stomatal kinetics under WS conditions, isohydric cultivars prevent major drops in Ψ_leaf_ by early stomatal closure whereas anisohydric cultivars maximize photosynthetic gain by keeping the stomata open despite significant decreases in Ψ_leaf_ [8]. However, grapevine cultivars demonstrate a range of responses between perfectly isohydric and anisohydric behaviors, and the two strategies can occur within the same cultivar depending on the environmental conditions. The hydraulic behaviors of different grapevine varieties are controversial [4, 9, 11, 12]. Water deprivation influences grapevine survival, growth and productivity [4, 13] as well as berry flavor and composition [14, 15]. Intracellular concentrations of the phytohormone abscisic acid (ABA) tend to increase in plants subjected to WS. The signal transduction cascade triggered by ABA, and involving ABA-induced gene expression, eventually leads to stomatal closure and thus water retention [16, 17]. ABA also acts in concert with other hormones to ensure a rapid and targeted response which is tightly regulated by a complex network of signaling pathways [18, 19].

Recently, large-scale gene expression analysis has been used to investigate the WS response in different grapevine tissues, improving our knowledge of transcriptional regulation during drought [15, 20–22]. These studies revealed the importance of genes controlling stress-related signaling cascades, those coding for proteins directly involved in the protection of membrane integrity, those implicated in water and ion uptake and transport, and those encoding heat-shock proteins (HSPs) and chaperones, late embryogenesis abundant (LEA) proteins, osmoprotectants and free radical scavengers. Interestingly, comparative transcriptomics analysis of WS responses in a red-berry variety (Cabernet Sauvignon) and a white-berry variety (Chardonnay) showed that water deficit increased ABA, proline, and sugar concentrations in Cabernet Sauvignon but not Chardonnay berries [15]. Two very recent studies used high-throughput RNA-seq analysis to investigate the impact of WS on rootstock genotypes [20, 21]. The selection and breeding of rootstock to improve water use efficiency is one of the key strategies that could be used to address climate change. These studies showed that WS induces many changes in secondary metabolism leading to the biosynthesis of secondary compounds in roots, leaves and also in the berries.

An important characteristic of non-irrigated crops in temperate climates and irrigated crops in arid climates is that they are continuously subjected to cycles of WS and re-watering. Rehydration induces the reversal of many WS effects, but the dynamics of these processes are different (e.g. Ψ_leaf_ increases rapidly whereas transpiration and photosynthesis recover more slowly) [23]. The recovery from WS in Grenache (a near-isohydric cultivar) has been recently assessed by microarray analysis of the leaf petiole transcriptome, showing that the gene categories most strongly affected were those involved in secondary metabolism, sugar metabolism and transport, as well as several aquaporin genes [24].

The current body of evidence suggests that the strict division of grapevine varieties into isohydric and anisohydric categories is at least premature and maybe inappropriate, and that more effort is needed to determine the relative importance of true genetic differences in stomatal control compared to variations that reflect scion/rootstock combinations, climate (temperature and air-to-leaf vapor pressure deficit, VPD), growing conditions and the extent and duration of WS. A number of cultivars have evolved complex strategies to cope in high-temperate environments and these call for an integrated approach to investigate the phenomena involved.

Here, we characterized variations in leaf biochemistry, gas exchange and energy dissipation mechanisms in potted grapevine plants of the varieties Sangiovese (near-anisohydric) and Montepulciano (near-isohydric) [25] subjected to a pre-veraison deficit irrigation, combined with a genome-wide expression analysis followed by multivariate analysis using different statistical approaches aiming to explore the effects of early water deficit on the leaf transcriptome. Furthermore, we explored the reactions of the leaf transcriptome in both cultivars after re-watering and studied the impact of WS on berry physiology and gene expression.

## Results

### WS imposition and sampling strategy

**Fig. 1:**
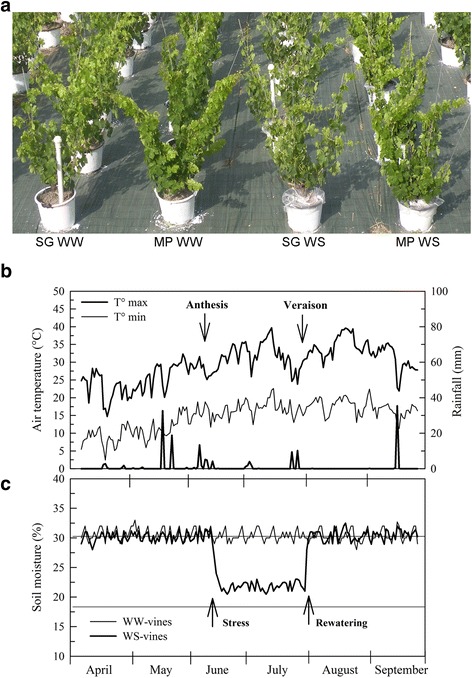
Design and environmental parameters of the water stress experiment. **a** vines used for physiological and transcriptomic analysis at berries pea-size phenological stage (end of June). Pots were covered with a plastic film during water limitation. SG = Sangiovese; MP = Montepulciano; WW = well-watered vines; WS = water-stressed vines. **b** Seasonal trends of maximum and minimum air temperature and rainfall. **c** Soil moisture measured in the pots in WW and WS vines

Montepulciano and Sangiovese potted vines were subjected to a water deficit at 40 % of maximum water availability from fruit-set to veraison (onset of ripening) and were then fully re-watered (Fig. 1a and c). As previously observed by Palliotti et al. [13], water stress conditions induced a faster and more pronounced basal leaf yellowing and shedding in Sangiovese than in Montepulciano vines. Daily minimum and maximum temperatures and rainfall were monitored during the experiment (Fig. 1b). Three pools of fully expanded leaves, sampled between nodes 14 and 16 of the primary shoots of well-watered (WW) and water-stressed (WS) vines, were sourced from both varieties 2, 6 and 27 days after WS was imposed, to assess the short and long-term effect of WS on vine physiology, and were used for the analysis of all physiological, biochemical and transcriptional characteristics.

### WS differentially affects leaf gas exchange and ABA content in Sangiovese and Montepulciano

**Fig. 2:**
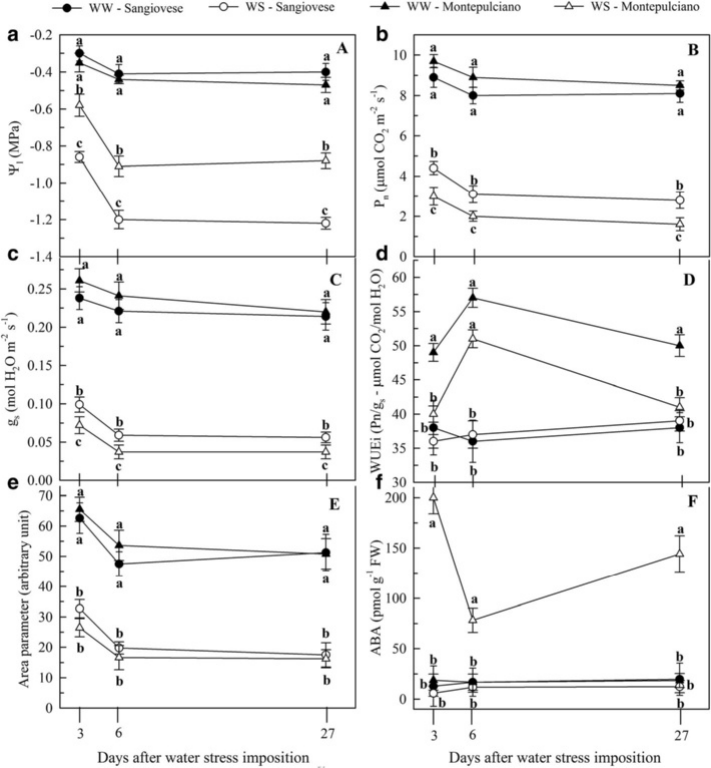
Dynamics of physiological parameters. Changes in leaf water potential (**a**), maximum net photosynthesis (**b**), stomatal conductance (**c**), intrinsic water use efficiency (**d**), *Area* parameter (**e**) and ABA content (**f**) for Sangiovese and Montepulciano vines under well-watered (WW) and water-stressed (WS) conditions. Data were taken 2, 6 and 27 days after WS. For each measurement date, the means ± SE followed by *different letters* are significantly different at *p* < 0.05 according to the Student-Newman-Keuls test

After 2 days of WS, the midday leaf water potential (Ψ_l_) fell to −0.86 MPa in the Sangiovese vines but to only −0.58 MPa in the Montepulciano vines (Fig. 2a). After 6 days of WS, the values fell to −1.20 and −0.91 MPa, respectively, and these values were maintained until 27 days after WS was imposed (Fig. 2a). A similar decreasing trend was observed for the net photosynthesis (A_max_) and stomatal conductance (g_s_) (Fig. 2b and c). Although the A_max_ and g_s_ rates of the WW vines of both cultivars did not change during the experiment, the WS Sangiovese leaves showed higher A_max_ and g_s_ values than WS Montepulciano leaves after 2, 6 and 27 days of WS (Fig. 2b and c). The WS Sangiovese vines also showed a significant increase in the intrinsic water use efficiency (WUE_i_) after 2 and 27 days of WS, whereas there was no increase in the WS Montepulciano vines (Fig. 2d). After 6 days of WS, the WUEi was significantly higher in both cultivars (Fig. 2d).

Regardless of the cultivar, the *Area* parameter was halved for the duration of WS, indicating a drastic reduction of the plastoquinone pool size on the reducing side of PSII (Fig. 2e). Conversely, the F_v_/F_m_, F_o_ and F_m_ parameters were unaffected in both cultivars during the experiment (Additional file 1). The ABA content of the WS Montepulciano leaves increased significantly compared to WW vines, whereas there was no change in the ABA content of the WS Sangiovese leaves compared to the WW controls (Fig. 2f).

### WS affects the leaf pigment content in Sangiovese and the de-epoxidation state in Montepulciano

The total chlorophyll content (Chl_total_) of the WS Sangiovese leaves increased significantly compared to the corresponding WW leaves, particularly reflecting an increase in the levels of Chl *a*, whereas the WS Montepulciano leaves showed no significant changes (Additional file 1).

The analysis of xanthophylls showed no significant variation in the levels of individual carotenoids, the de-epoxidation state (DEPS) or the Car_total_/Chl_total_ ratio in either cultivar after 2 days of WS (Additional file 1). However, after 6 days the WS Sangiovese leaves showed a significant increase in the violaxanthin, antheraxanthin and zeaxanthin (VAZ) pool and total carotenoids (Car_total_) compared to WW leaves (Additional file 1), due to the accumulation of β-carotene, lutein, antheraxanthin and violaxanthin. The WS Montepulciano leaves showed a significant increase in the DEPS compared to the corresponding WW vines, due to the loss of violaxanthin but the simultaneous accumulation of zeaxanthin (Additional file 1).

After 27 days, the Car_total_ and VAZ pool increased significantly in WS Sangiovese leaves due to the accumulation of antheraxanthin, zeaxanthin, neoxanthin, lutein and β-carotene (Additional file 1). Although the DEPS increased by almost 100 % in both cultivars under WS, the Montepulciano cultivar nevertheless achieved full activation of the de-epoxidation process due to the loss of violaxanthin and a concomitant increase in the levels of zeaxanthin and antheraxanthin (Additional file 1).

Finally, the WS Montepulciano leaves displayed a significant increase in both H_2_O_2_ concentration and catalase (CAT) activity during the WS period in comparison to the WW vines but no such changes were observed in WS Sangiovese leaves (Additional file 1).

### Whole genome transcriptional analysis in leaves subjected to WS

**Fig. 3:**
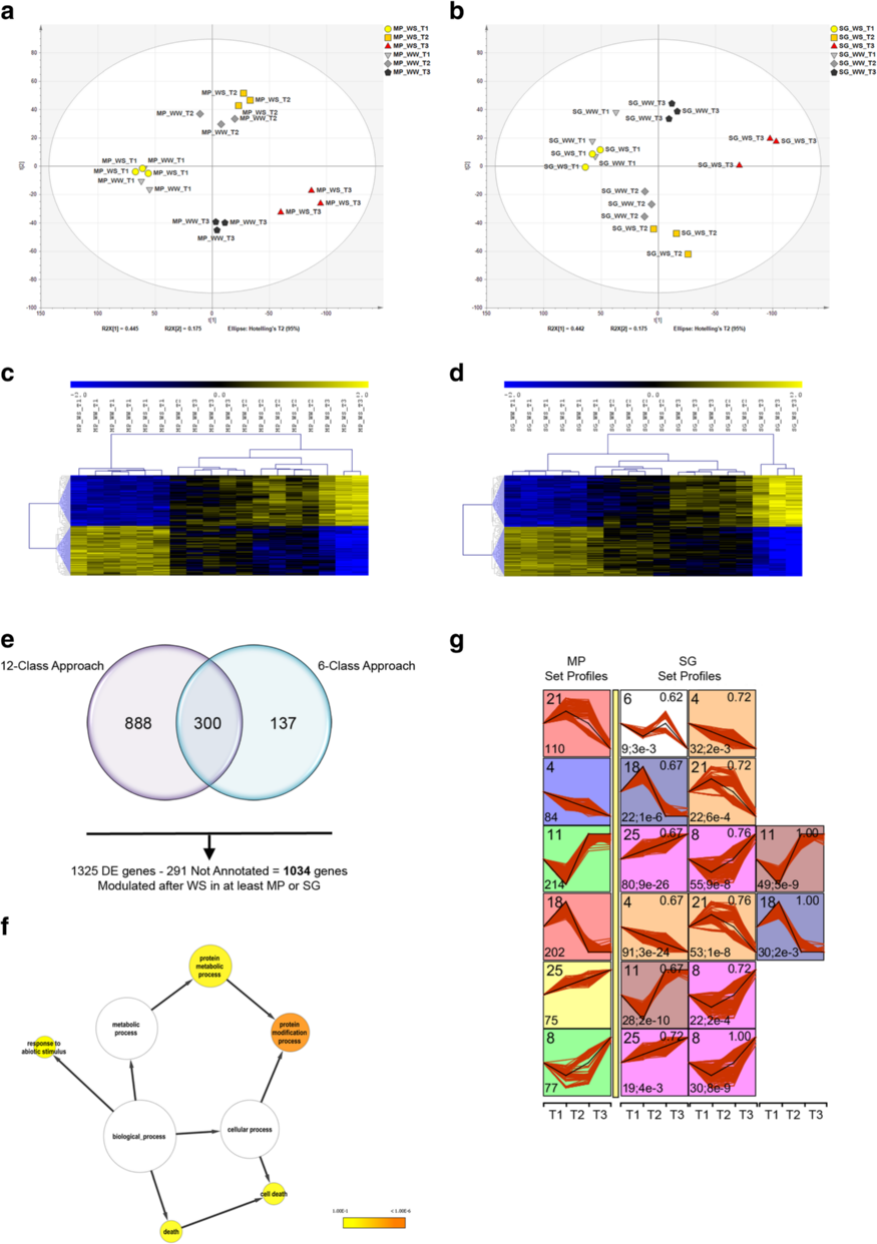
Whole genome transcriptional analysis in Montepulciano (MP) and Sangiovese (SG) leaves subjected to water stress (WS). PCA and heat-map representation of the PCA loadings (first and last percentile) shows the significantly modulated genes in MP (**a**, **c**) and SG (**b**, **d**) vines under well-watered control (WW) and WS conditions. Expression was measured as the log_2_ intensity of each biological replicate. Each value was normalized on the median value of each row/gene and Euclidean’s correlation distance was used as the metric. **e** Venn diagram summarizing the differentially expressed genes retrieved by distinct approaches (see text). **f**, Enriched GO terms for the 1034 stress-modulated genes. The network graphs show BiNGO visualizations of the overrepresented GO terms. Categories in GoSlimPlants [66] were used to simplify this analysis. *Colored nodes* represent GO terms that are significantly overrepresented (*p* < 0.1). **g** Significant profiles (<5 % Bonferroni correction method) of the 1034 modulated genes during WS, from among 30 profiles subjected to STEM analysis, in MP (*left column*) and SG leaves (to the right of the *yellow bar columns*). In each frame, the number of genes shown in each profile is displayed to the bottom left whereas the number ID for each profile (from 1 to 30) is shown to the top left. *Red curves* represent individual profiles (i.e. profile of the fold change between WW and WS conditions) and the *black line* represents the profile to which they are most similar. Significant expression profiles are highlighted in color wherein the same background color represents a similar profile. Frames of the compared set profile (SG, to the right of the *yellow bar*) also show the comparison statistics. The correlation between MP and SG profiles is shown to the upper right as a number (1.00 indicates the same profile). The number of genes assigned to the MP profile that were also assigned to the SG profile is shown at the bottom left, followed by an indication of the *p*-value for the number of genes at the intersection. See Additional file 4 for the complete comparison profile table. The x-axis represents sampling points and the y-axis denotes log_2_ scale fold change in expression

The leaf transcriptome data set of both varieties after 2, 6 and 27 days of WS was initially screened by significance analysis of microarrays (SAM, 12 groups, FDR = 0.1 %) to select genes that were differentially modulated under our experimental conditions (18,413 genes). Analysis of variance (ANOVA, 12 groups, α = 0.01, standard Bonferroni correction) was applied to transcripts positive in the previous SAM experiment in order to skim off the most significantly modulated transcripts (5947 genes, Additional file 2). A PCA was used to verify the consistency of biological replicates and to generally inspect the transcriptomes of the Montepulciano and Sangiovese varieties under WS (Fig. 3a and b). In both PCA plots, PC1 explained ~44 % of the total data set variability and mostly reflected differences among the three sampling points and, within a single sampling point, differences between the WW and WS samples in both varieties. PC1 loadings clearly showed that the dynamics of leaf stress responses are different in the two varieties (Fig. 3c and d). Indeed, the reaction of the Montepulciano transcriptome towards WS started at the second sampling point and continued in the third with gradually increasing intensity, whereas the Sangiovese transcriptome began to react only at the third sampling point, albeit with a stronger shift (Fig. 3c and d).

To evaluate differences in gene expression between Montepulciano and Sangiovese under WS, we focused on changes in expression profiles of genes scoring a fold change (FC) ≥2 between the WW and WS vines of each variety at each sampling point. We identified 1188 genes using this approach (Fig. 3e, Additional file 3). To identify further genes modulated by WS*,* the same statistical procedure was followed but this time considering only the WS and WW categories regardless of the genotype (six-class SAM and ANOVA). We found 437 modulated genes shared between Montepulciano and Sangiovese, 300 of which (~67 %) were already present among the 1188 genes identified in the initial statistical strategy (Fig. 3e). Hence, we found a total of 1325 genes modulated by WS in at least one of the two cultivars, among which 1034 were functionally annotated (Fig. 3e, Additional file 3). Overall, these 1034 stress-modulated genes were particularly enriched, as expected, in functional categories related to stress such as “Response to abiotic stimulus”, “Death” “Cell death” and “Protein metabolic and modification process” (Fig. 3f).

Differences in the response to WS between Montepulciano and Sangiovese were investigated in more detail by applying the Short Time-series Expression Miner (STEM) clustering method [26] to the 1034 stress-modulated genes. This enabled us to visualize groups of genes whose differential expression between WS and WW samples also differed significantly between the two genotypes (Additional file 4). Figure 3g shows that some Montepulciano transcripts, clustered accordingly with their expression profiles (right side), displayed a significantly different expression profile in the Sangiovese cultivar (left side).

#### ABA-related genes

**Fig. 4:**
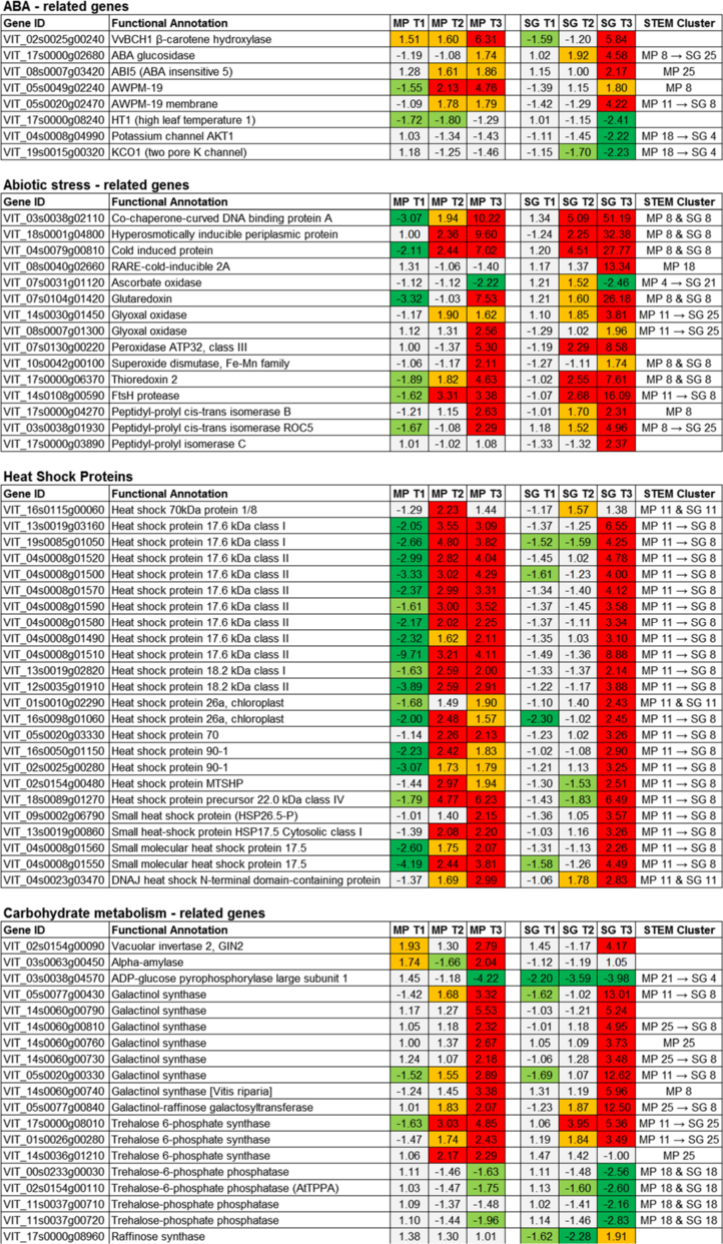
Selected genes affected by water stress (WS) in the leaves of Montepulciano (MP) and/or Sangiovese (SG) vines. The STEM cluster column reports STEM cluster number as in Fig. 3g. The *arrow* → indicates a cluster swapping between MP and SG whereas the letter & indicate a cluster retaining

Many ABA-related genes modulated by WS were differentially expressed in the leaves of the two varieties, including β-carotene hydroxylase VvBCH1 (VIT_02s0025g00240) which was induced from the onset of WS in Montepulciano but only after 27 days in Sangiovese, and an ABA glucosidase (VIT_17s0000g02680) which was induced 6 and 27 days after the onset of WS in Sangiovese but only after 27 days in Montepulciano (MP 8 → SG 25 in STEM analysis, Figs. 3g and 4). The ABA-responsive bZIP transcription factor VvbZIP25 (ABA Insensitive 5, VIT_08s0007g03420) [27] was upregulated in both varieties but more rapidly in Montepulciano (Fig. 4). Two transcripts encoding membrane proteins of the AWPM-19-like family (VIT_05s0049g02240 and VIT_05s0020g02470) whose levels dramatically increase when the intracellular concentration of ABA increases [28], were upregulated after 6 days of WS in Montepulciano leaves but only after 27 days in Sangiovese (MP 11 → SG 8 in STEM analysis, Figs. 3g and 4). These data agree with the significant increase in the H_2_O_2_ accumulation and CAT activity in WS Montepulciano leaves at any sampling point compared to WW controls (Additional file 1). The Sangiovese leaves showed no differences in H_2_O_2_ levels or CAT activity under WS (Additional file 1). The main negative regulator of the stomatal closure pathway, HT1 (High leaf Temperature 1, VIT_17s0000g08240) [29] was repressed after 2 and 6 days in Montepulciano, but only after 27 days in Sangiovese leaves.

#### Abiotic stress-related genes

WS triggered abiotic stress-related transcriptional responses in the leaves of both cultivars, but these genes tended to be regulated more strongly in Sangiovese (Fig. 4). Genes encoding dehydrins, osmotines, thaumatins, chaperones, cold-induced proteins and senescence-associated proteins were strongly upregulated. Eleven genes involved in ROS scavenging during the oxidative burst [30] were upregulated in both cultivars, including those encoding ascorbate peroxidase, glutaredoxin, peroxidase, glyoxal oxidase, peptidil-prolyl-trans isomerase and thioredoxin. Genes involved in the oxidative stress-induced protein damage repair pathway [31] were more strongly induced in Sangiovese leaves. Twenty-four heat shock and heat shock-related proteins (HSPs) were differentially modulated in each variety, although they were upregulated after 27 days in Sangiovese but suppressed after 2 days and upregulated from 6 days onwards in Montepulciano (MP 11 → SG 8 in STEM analysis, Figs. 3g and 4).

#### Carbohydrate metabolism-related genes

Drought-stressed plants accumulate a large amount of water-soluble carbohydrates, which are used as osmolytes to maintain leaf cell turgor, protect membrane integrity and prevent protein denaturation [32]. The vacuolar invertase *VvGIN2* (VIT_02s0154g00090) was induced in Montepulciano at the onset of WS but was delayed in Sangiovese (Fig. 4). The starch-degrading enzyme α-amylase (VIT_03s0063g00450) was induced in Montepulciano but not Sangiovese, whereas ADP-glucose pyrophosphorylase (VIT_03s0038g04570) was downregulated from the first sampling point in Sangiovese but only at the last sampling point in Montepulciano (MP21 → SG4 in STEM analysis, Figs. 3g and 4). Seven galactinol synthases and one galactinol-raffinosegalactosyl transferase were upregulated in both varieties, but more strongly in Sangiovese (MP25 → SG8 in STEM analysis, Figs 3g and 4). Three trehalose-6-phosphate synthases were upregulated and four trehalose-6-phosphate phosphatases were downregulated in both cultivars (Fig. 4). Finally, a raffinose synthase (VIT_17s0000g08960) was downregulated only in Sangiovese at the first and second sampling points but was induced at the third sampling point, whereas no variation in transcript levels was detected in Montepulciano leaves indicating that the type of water-soluble carbohydrates that accumulate during WS can vary among different cultivars.

### Switch genes are putative negative biomarkers of WS

**Fig. 5:**
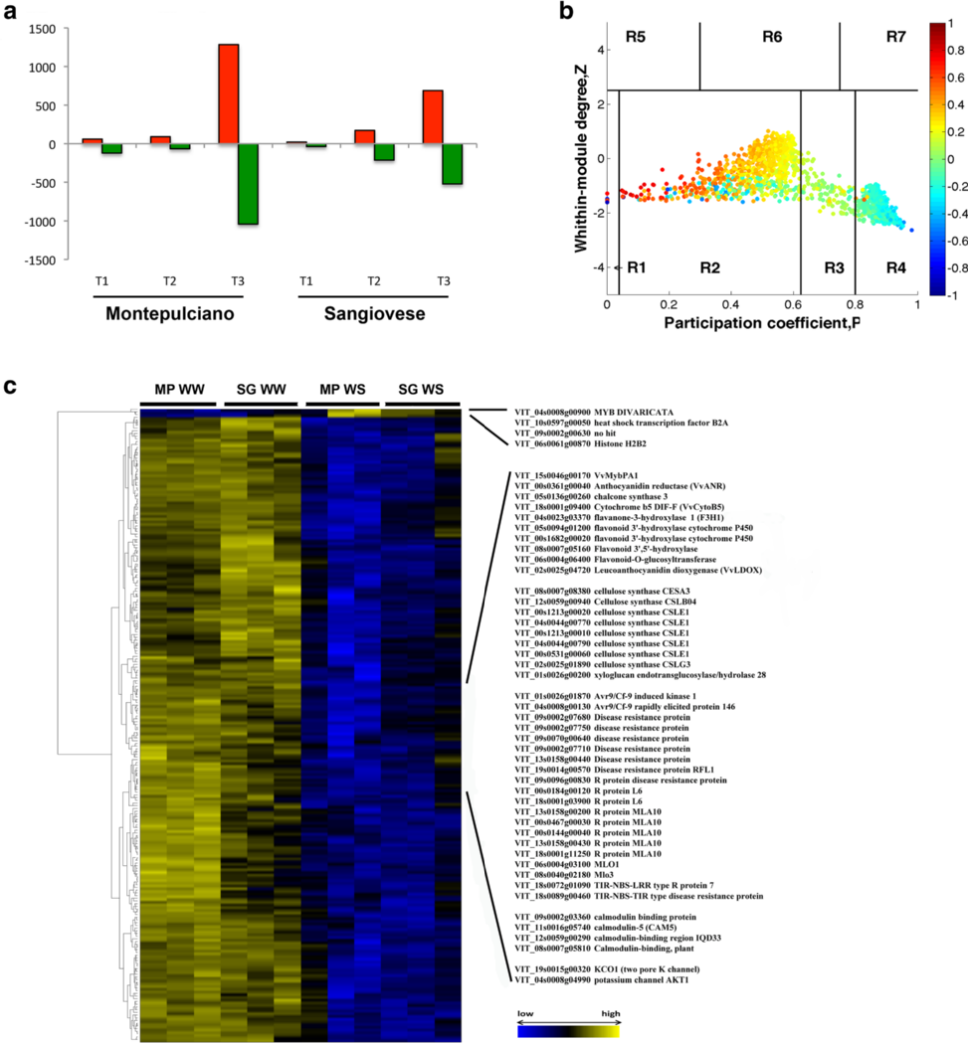
Putative negative biomarkers (switch genes) of WS in grapevine. **a** Genes that are differentially expressed between WW and WS leaves at each time point in both genotypes following the application of a *t*-test (*p* < 0.01) and a fold change filter (|FC| ≥2 between WS and WW leaves). **b** Heat cartography map. The plane identified by the two parameters Z and P is divided into seven regions each defining a specific node role. Each point represents a node in the correlation network in Additional file 7, and the color of each node corresponds to its average Pearson correlation coefficient value. Roles were assigned to each node in the correlation network according to the heat cartography. **c** Heat map representing the 298 switch genes representing putative key regulators of the transcriptome shift from WW to WS status in grapevine leaves. Expression was measured as the log_2_ intensity of each biological replicate. Each value was normalized on the median value of each row/gene and Pearson’s correlation distance was used as the metric

In order to identify putative molecular markers of WS in grapevine, we collected the genes that are differentially expressed between WW and WS leaves at each sampling point for each genotype separately and then applied a *t*-test (*p* < 0.01) and filtered the genes with a FC ≥2 when WS and WW leaves were compared. We found that both genotypes were characterized by a small number of differentially expressed genes at the first two sampling points compared with the third point (Fig. 5a and Additional file 5). After 2 days of WS, 181 genes were differentially expressed in Montepulciano leaves and only 59 in Sangiovese leaves. Interestingly, at this time point, both cultivars were characterized by a higher number of downregulated rather than upregulated genes, and only mitogen-activated protein kinase kinase kinase 15 (MAPKKK15; VIT_10s0116g01230) was downregulated in both cultivars. The higher number of differentially expressed genes in Montepulciano leaves provides evidence that this cultivar responds to WS more quickly than Sangiovese at the transcriptomic level, as also revealed by PCA (Fig. 3a–d). After 6 days of WS, 156 genes were differentially expressed in Montepulciano and 386 in Sangiovese. At this time point, there were more downregulated than upregulated genes in Sangiovese leaves (214 vs. 172), but the opposite trend was apparent in Montepulciano leaves (66 vs. 90).

By the third sampling point (after 27 days of WS) there were more upregulated than downregulated genes in both cultivars. Interestingly, the upregulated genes were more strongly induced in Sangiovese compared to Montepulciano leaves, whereas the downregulated genes were more strongly suppressed in Montepulciano compared to Sangiovese leaves. This suggests that the slower response of Sangiovese leaves to WS is balanced by the stronger induction of the response genes. We found 169 differentially expressed genes shared between the two cultivars (Fig. 5a and Additional file 5), suggesting that responses to WS become more aligned between the cultivars at the final sampling point.

WW and WS leaves from each cultivar after 27 days of WS were next analyzed using our recently published integrated approach based on topological co-expression networks to identify common putative key regulators of WS [33]. A comparison of the WW and WS leaf transcriptomes regardless of genotype revealed 1236 genes that are differentially expressed between WW and WS leaves (*p* < 0.08; FC >1.7). We found that 765 genes were upregulated and 471 were downregulated under WS (Additional file 6), confirming that 27 days of WS predominantly causes gene activation rather than suppression. The coexpression network, based on Pearson correlations, comprised 1236 nodes and 202,422 edges (Additional file 7). By applying the date/hub classification system to define the topological proprieties of the network, we identified 405 Fight-club hubs and 298 switch genes (Fig. 5b and Additional file 6). As previously reported [33], switch genes are characterized by a pronounced negative correlation with the expression profiles of neighboring genes outside their own group in the network, and therefore represent putative key regulators of leaf transcriptome remodeling during the shift from the WW to the WS environment. Interestingly, we found only four genes expressed at low levels in WW leaves but upregulated in WS leaves, whereas most of the switch genes were downregulated in WS leaves. Among the four upregulated genes, we identified heat shock transcription factor B2A (VIT_10s0597g00050), histone H2B2 (VIT_06s0061g00870) and the MYB floral symmetry gene *DIVARICATA* (VIT_04s0008g00900) whereas the remaining gene did not provide a match (VIT_09s0002g00630) (Fig. 5c).

Among switch genes downregulated in WS leaves we found many representing the flavonoid biosynthesis pathway, including *VvMYBPA1* (VIT_15s0046g00170) and its target *VvANR* (VIT_00s0361g00040), *VvCHS3* (VIT_05s0136g00260), Cytochrome b5 DIF-F (VvCytoB5; VIT_18s0001g09400), and a flavonoid 3′-5′-hydroxylase (VIT_08s0007g05160). We also found genes related to cell wall metabolism, including cellulose synthases and a xyloglucan endotransglucosylase/hydrolase, and many genes related to biotic stress responses, such as R protein, Avr9/Cf-9 induced kinase and a TIR-NBS-TIR type disease resistance protein. Interestingly, four calmodulin proteins were identified among the switch genes, including CAM5 (VIT_11s0016g05740), which is downregulated in response to heat stress in Arabidopsis [34]. Finally, the two-pore potassium channel KCO1 and the AKT1 channel, described, were also found among the switch genes which are downregulated in WS leaves (Fig. 5c and Additional file 6).

### Characterization of the physiology and transcriptome of leaves following stress recovery

After 46 days of WS, the vines were re-watered and allowed to return to ~90 % of maximum water availability (Fig. 1c) which was achieved after another 24 days. Leaves were sampled from the WW plants, which had received an uninterrupted water supply throughout the experiment, and the revived WS plants (RWS).

**Fig. 6:**
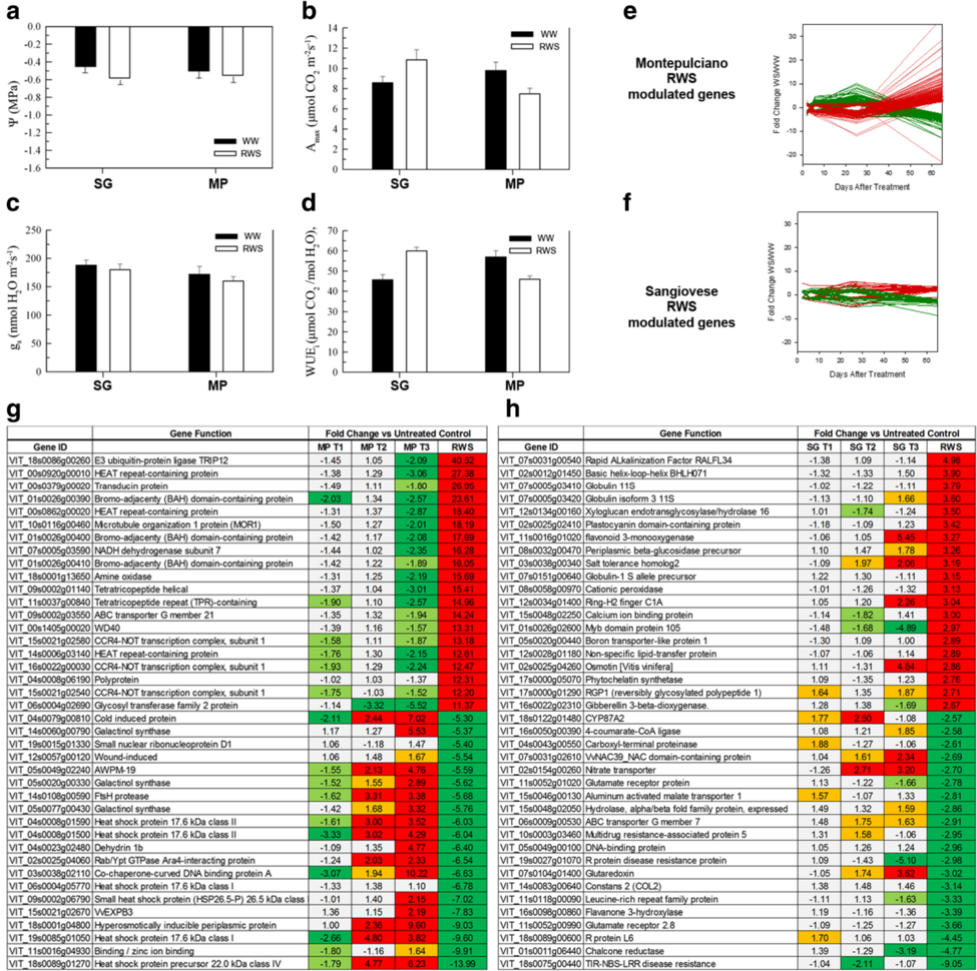
Characterization of recovery from water stress (RWS) in Montepulciano (MP) and Sangiovese (SG) leaves. **a**–**d** Photosynthesis parameters of SG and MP vines under well-watered control conditions (WW) and after recovery from water stress. Data were recorded when the RWS vines reached ~90 % of maximum water availability. **e**–**f** Line plots of the differentially modulated genes exhibiting a Fold Change (FC) ≥3 or ≤−3 between RWS and WW samples, throughout the entire treatment period in MP and SG, respectively. **g**–**h** Selected genes modulated during RWS in MP and in SG leaves, respectively. The differentially modulated genes between RWS and WW samples were FC ranked and the top 20 and bottom 20 annotated genes are shown in the charts

Physiologically, the two cultivars behaved differently after rehydration (Fig. 6a–d). Although both cultivars promptly recovered to a non-limiting Ψ_l_ (approximately −0.6 MPa) (Fig. 6a), the RWS Sangiovese leaves reached a higher A_max_ value than corresponding WW vines, whereas RWS Montepulciano leaves only achieved a partial A_max_ recovery, setting at 79 % of the corresponding WW vines (Fig. 6b). The g_s_ rates of RWS leaves from both cultivars returned to values similar to WW vines (Fig. 6c). Therefore, differences in A_max_ following rehydration were also reflected in the WUE_i_ values, which were significantly higher than WW controls in RWS Sangiovese leaves but significantly lower than WW controls in RWS Montepulciano leaves (Fig. 6d).

Re-watering also had a differential impact on the transcriptomes of the two cultivars. Far more genes were differentially expressed between WW and RWS leaves in the Montepulciano vines (2381 genes) compared to Sangiovese vines, where only 197 were identified (Fig. 6e and f; Additional file 8). There was also a greater FC between WW and RWS gene expression levels in Montepulciano leaves compared to Sangiovese leaves (Fig. 6g and h). The majority of the Montepulciano transcripts modulated by RWS reversed the expression profiles observed during WS, i.e. transcripts downregulated by WS were upregulated by re-watering and vice versa (Fig. 6e and h), whereas this reversal was not observed in the Sangiovese leaves (Fig. 6f). Interestingly, many of the genes most strongly induced by recovery in the Montepulciano leaves were involved in protein regulatory activities, suggesting that adjustments following the release of stress are achieved predominantly through the management of existing protein pools. Nine HEAT-repeat-containing proteins mainly involved in cargo transport and in protein translation [35] were strongly upregulated in the Montepulciano RWS leaves, as well as three bromo-adjacent homology (BAH) domain-containing proteins and six CCR4-NOT transcription factors, both with roles in gene expression regulation [36]. Six E3 ubiquitin protein ligases with a well-known role in protein degradation [37] were also upregulated in Montepulciano RWS leaves. Many transcripts for HSPs and galactinol synthases that were induced by WS were among the most strongly suppressed genes in the RWS Montepulciano leaves. Taken together, these results indicate that the recovery process in Montepulciano leaves actively counteracts the negative effects of prolonged WS, whereas there is no equivalent process in Sangiovese leaves (Fig. 6h).

### Whole genome transcriptional analysis in berries subjected to WS

We also compared the transcriptome dynamics of Montepulciano and Sangiovese berries sampled at the same time as the leaves. These sampling points span the herbaceous growth phase, corresponding to BBCH 69 (end of flowering with all flowerhoods fallen), BBCH 71 (fruit set: young fruit begin to swell, remains of flower lost) and BBCH 77 (berries begin to touch) as described by Lorenz et al. [38].

**Fig. 7:**
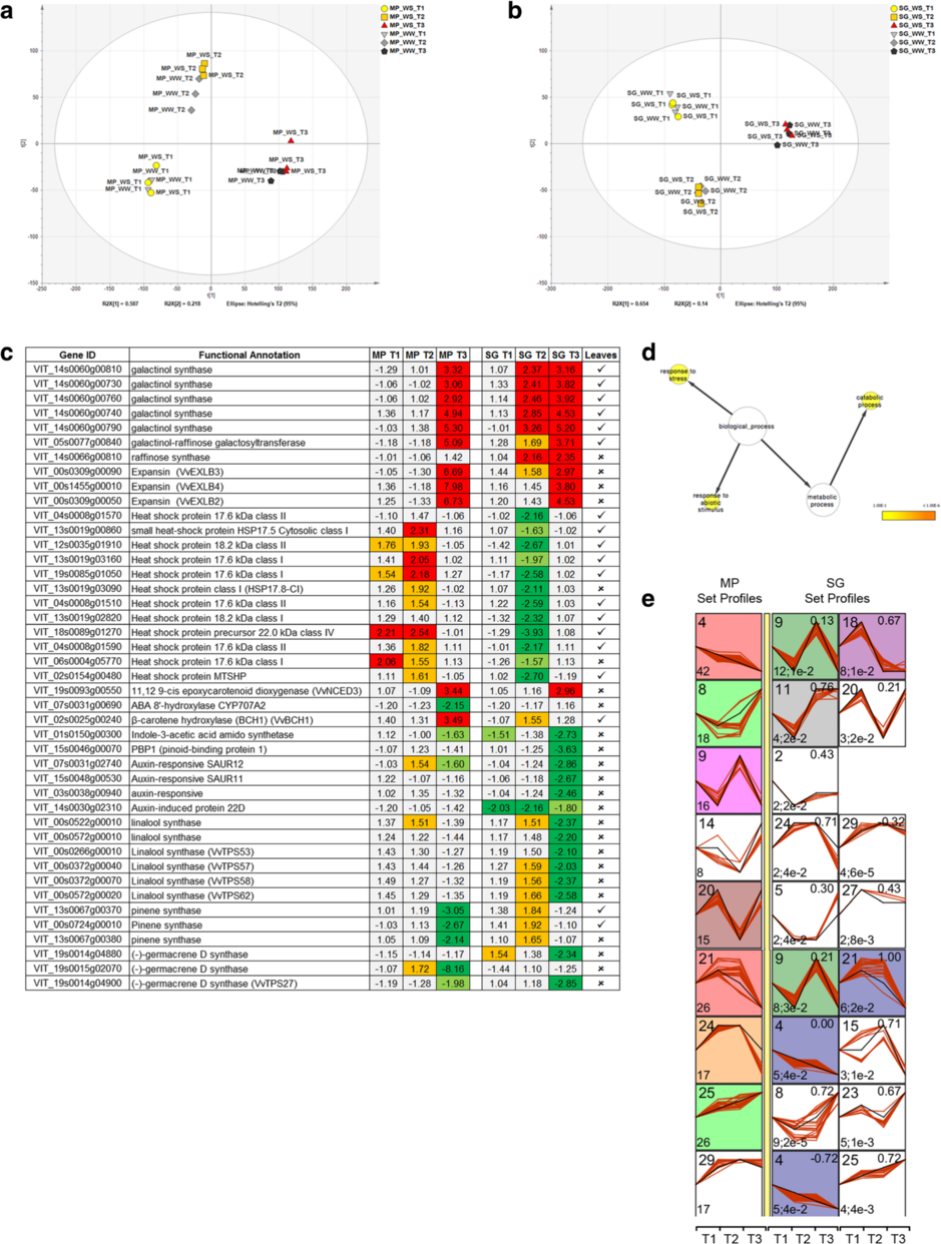
Whole genome transcriptional analysis in Montepulciano (MP) and Sangiovese (SG) berries subjected to water stress (WS). **a**–**b** PCA of the significantly modulated genes under well-watered control (WW) and WS conditions in MP and in SG vines, respectively. **c** Selected genes affected in berries under WS in MP and/or SG vines. The presence or the absence of the same gene in the leaf data set is denoted by the ✓ and ✗ symbols, respectively. **d** Enriched GO terms for the 269 stress-modulated genes. The network graphs show BiNGO visualizations of the overrepresented GO terms. Categories in GoSlimPlants [66] were used to simplify this analysis. Colored nodes represent GO terms that are significantly overrepresented (*p* < 0.1). e Significant profiles (<5 % Bonferroni correction method) of the 269 modulated genes during WS, from among 30 profiles subjected to STEM analysis, in MP (*left column*) and SG leaves (to the right of the *yellow bar columns*). For more details on STEM analysis, refer to Fig. 3g legend

As per transcriptomic analysis, the same statistical workflow as applied to the leaves was applied to the berries. SAM (12 groups, FDR = 0.1 %) revealed that 23,464 genes were differentially modulated under our experimental conditions, and ANOVA (12 groups, α = 0.01, standard Bonferroni correction) retrieved the 11,839 most significantly modulated transcripts (Additional file 9). Genotype-specific PCA (Fig. 7a and b) revealed consistency among the biological triplicate samples. This statistical approach also suggested that most of the data set variability explained differences among the phenological stages and not between the WW and WS samples.

We identified 354 differentially expressed berry genes by comparing WW and WS plants (FC ≥2) at each time point in each variety, among which 269 were already annotated (Fig. 7c and Additional file 10). These transcripts, 48 (~18 %) of which were also found among the stress-modulated transcripts in the leaves, were particularly enriched in the functional categories “Response to stress”, “Response to abiotic stimulus” and “Catabolic process”, as highlighted by the BiNGO overrepresentation analysis (Fig. 7d). Overall, the response of berries to WS involved fewer genes than the leaves, and these genes were generally subject to weaker modulation (Additional file 10).

We next used the STEM clustering approach to determine the extent to which the WS response differs between berries of the two varieties (Fig. 7e and Additional file 11). Five galactinol synthases, a galactinol-raffinose galactosyltransferase and a raffinose synthase were upregulated more strongly and rapidly in Sangiovese compared to Montepulciano berries (SG25 → MP8 and SG25 → MP23 in STEM analysis, Fig. 7c and e). With the exception of the raffinose synthase (VIT_14s0066g00810), these genes were also expressed in WS leaves (Additional file 10). Three β-expansin-like genes (VvEXLB2–4) were upregulated by WS but the profile differed between the cultivars (SG25 → MP8 and SG25 → MP23 in STEM analysis, Fig. 7c and e). Ten of the 12 of heat shock and heat shock-related proteins were also commonly expressed in berries and leaves but the expression profiles were distinct (Fig. 7c and Additional file 10). In the berries, these genes were downregulated after 6 days in Sangiovese vines and almost no modulation was observed at the other time points or in the Montepulciano berries (SG14 → MP29 in STEM analysis, Fig. 7c and e). The difference between the varieties in terms of heat management in the leaves therefore appeared to be lost in the berries.

**Fig. 8:**
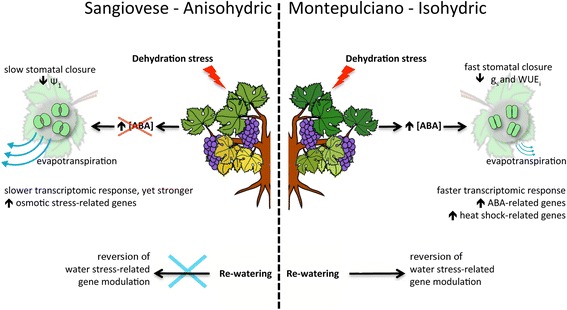
Schematic representation of the distinct responses to water limitation in Sangiovese and Montepulciano. g_s_ = stomatal conductance; WUE_i_ = intrinsic water use efficiency; Ψ_1_ = leaf water potential; ABA = abscisic acid

Three important ABA-related transcripts were significantly modulated in berries subjected to WS. The 11,12,9-cis epoxycarotenoid dioxygenase VvNCED3 (VIT_19s0093g00550), which catalyzes the last step in ABA biosynthesis [39], was upregulated in both varieties albeit with minor differences in the expression profile (SG 25 → MP 23 in STEM analysis, Fig. 7c and e; Additional file 11). The β-carotene hydroxylase VvBCH1, which was expressed in WS leaves, was upregulated at the final sampling point in Montepulciano berries but was not modulated in Sangiovese berries. Furthermore, the ABA-degrading enzyme ABA 8′-hydroxylase CYP707A2 (VIT_07s0031g00690) was downregulated at the final sampling point in Montepulciano berries but not in Sangiovese berries. These findings suggest that ABA is synthesized in berries after prolonged WS in both varieties, but only in Montepulciano berries is the ABA level maintained by downregulating the enzyme responsible for ABA degradation. Interestingly, genes related to auxin metabolism and signal transduction were more strongly repressed in Sangiovese than Montepulciano berries, e.g. indole-3-acetic acid amidosynthetase (VIT_01s0150g00300), (SG4 → MP18 in STEM analysis, Fig. 8c and e).

Finally, the metabolism of volatiles was remarkably impaired in the berries of both varieties under WS, although the volatiles that were affected differed in each cultivar. Six linalool synthases were downregulated in Sangiovese berries whereas three pinene synthases were repressed in Montepulciano berries. Furthermore, two germacrene-D-synthases (VIT_19s0014g04880 and VIT_19s0014g04900) were downregulated solely in Sangiovese berries but another (VIT_19s0015g02070) was strongly downregulated only in Montepulciano berries (Fig. 7c).

## Discussion

We conducted a physiological and genome-wide transcriptional comparative study of the behavior of two grapevine cultivars towards WS, the anisohydric Sangiovese, traditionally the most widespread vine in Tuscany, and the isohydric Montepulciano, widely planted throughout central and southern Italy. Both cultivars were grafted on the same rootstock (1103 Paulsen) to avoid the well-known rootstock effect on scion performance under WS [40].

Sangiovese showed a higher transpiration at similar leaf water potential (Ψ_1_) values. As this cultivar shows early basal leaf yellowing and drop upon WS, we could rule out the hypothesis of an increased transpiration due to a higher leaf area and confirm that this could be caused by a smaller loss of hydraulic conductivity due to lower xylem vulnerability to cavitation than Montepulciano, as elsewhere reported [13, 25]. As matter of fact the early basal leaf yellowing recorded in Sangiovese did translate into lower final primary leaf area while the same response is not at all seen in terms of laterals (Additional file 1).

The Montepulciano transcriptome showed evidence of global remodeling after 6 days of WS, whereas the response of the Sangiovese transcriptome was delayed, yet stronger. Interestingly, the decline in Ψ_1_ commences after 2 days of WS in both cultivars but was more severe in Sangiovese leaves. This suggests that the transcriptomic response to drought is more dependent to genotype than to the intensity of Ψ_1_ reduction.

ABA-induced stomatal closure, is a very well-known process in WS plants [17, 41]. Under our experimental conditions, Montepulciano leaves under WS accumulated ABA more rapidly than WW leaves, especially after 2 days, when the leaf ABA content was 10 times higher in WS leaves than WW leaves. In contrast, ABA levels did not increase significantly in Sangiovese leaves throughout the drought period. The faster stomatal closure in the isohydric Montepulciano cultivar was confirmed by the lower stomatal conductance (g_s_). However, because WS reduced the A_max_ proportionally more than g_s_ in the Montepulciano leaves, WUE_i_ was also lower in Montepulciano than Sangiovese leaves under WS [13, 42]. The anisohydric cultivar Sangiovese also reached more negative midday leaf water potential values than the isohydric cultivar Montepulciano. The net concentration of ABA in any particular tissue is determined by the rate of its biosynthesis, catabolism (degradation and conjugation) and transport [43]. It is therefore difficult to correlate ABA levels and ABA-related transcripts precisely. Even so, we found that ABA-related genes were modulated by WS earlier in Montepulciano than in Sangiovese leaves, revealing a positive relation among the biochemical, physiological and transcriptomic data.

We found many differentially modulated transcripts required for the management of ROS and excess temperature in the WS leaves. Generally, ROS-scavenging enzymes, molecular chaperones and abiotic-stress related genes were induced earlier and more strongly in the Sangiovese leaves potentially to reduce the damage caused by WS. These proteins also induce stronger resistance to the photo-inhibition phenomenon reported in the medial leaves of both potted and open-field Sangiovese vines during the hottest hour of summer days [13]. The faster stomatal closure in Montepulciano leaves delays the production of ROS and the limited evaporative cooling may cause the leaves to heat up [44, 45]. In this regard, our data show that the H_2_O_2_ content of Montepulciano leaves increased soon after the onset of WS followed by a significant increase in CAT activity. This implies that ROS production in Montepulciano leaves plays a significant role in the photoprotection of PSII. On the other hand, the accumulation of H_2_O_2_ below the toxic threshold induces the expression of defense-related genes and acts as a regulator of cellular activities [46]. H_2_O_2_ in plants may originate from photorespiration in the peroxisomes, and studies of different grapevine cultivars have shown that the photorespiration rate (P_r_) is cultivar-dependent [42]. Indeed, Palliotti et al. [47] found that P_r_, expressed as percentage of A_max_, increased under severe drought stress at a faster rate in Montepulciano than Sangiovese (i.e. 73 % vs. 52 %).

In all species, heat stress induces the production of HSPs [48] which act as molecular chaperones to prevent protein aggregation and enzyme inactivation at elevated temperatures [49]. HSPs are induced more rapidly in Montepulciano leaves than Sangiovese leaves under WS, suggesting that Montepulciano leaves are exposed to heat stress due to the stomatal closure at the earliest sampling point.

Plants under WS also undergo metabolic changes, including the production of compatible solutes that stabilize proteins, maintain cell turgor, and remove ROS [50]. Several genes related to carbohydrate metabolism were differentially expressed in the leaves of both varieties after 27 days of WS, suggesting that the synthesis and mobilization of soluble sugars in grapevine is a key strategy to cope with prolonged stress and the enhanced risk of osmotic imbalance.

Trehalose is synthesized in a two-step process involving trehalose-6-phosphate synthase (TPS) and trehalose-6-phosphate phosphatase (TPP) [51]. WS induced the expression of several TPSs and repressed the expression of one TPP in the leaves of both varieties, suggesting that trehalose-6-phosphate levels may increase in response to WS. The overexpression of trehalose biosynthesis genes ruled out a threalose direct protective role [52–54]. However, trehalose and pathway intermediates may regulate stress signaling [55], so the accumulation of trehalose-6-phosphate in both cultivars may facilitate WS perception and activate stress resistance mechanisms.

In contrast, raffinose family oligosaccharides (RFOs) accumulate to high levels in the leaves of many plant species under stress [56, 57]. Seven galactinol synthases and other RFO biosynthesis genes were induced by WS in Sangiovese and Montepulciano leaves, indicating that the accumulation of compatible solutes is used as a common strategy by Montepulciano and Sangiovese to cope with WS.

ABA-related genes, HSPs and carbohydrate-related genes were common to our data sets and previously published WS leaf data sets representing two different rootstocks genotypes, i.e. M4 and 101.14 [21], with 21.28 % shared genes, and leaves of Cabernet Sauvignon [22], with 12.53 % shared genes. These comparisons suggest that the adaptation to WS involves mechanisms that are likely to be shared among all grapevine genotypes.

Berries and leaves from both varieties upregulated the same galactinol synthases and raffinose synthases under WS, particularly the Sangiovese berries. HSPs were also among the differentially regulated transcripts common to WS berries and leaves. On the other hand, berry-specific stress-modulated genes included those encoding cell wall remodeling proteins, auxin response proteins and enzymes required for the synthesis of volatile metabolites. These data indicate that WS is perceived in berries, although the response is less robust than in leaves, and that both cultivars have shared and cultivar-specific WS-response strategies in their sink and source organs. While final grape composition was not specifically measured in this study, in the collateral paper by Palliotti et al. [13] where the same treatments were imposed, it was clearly shown that, despite a similar yield reduction under WS (~30 %), Sangiovese showed better performances in terms of relative °Brix reduction (−1.4° in SG *vs −*2.8° in MP) and, especially, when evaluated as total anthocyanins and phenols concentrations which were unchanged in WS whereas showing a significant reduction in Montepulciano.

The identification of putative molecular biomarkers of the WS response in grapevine leaves revealed that transcriptomic reactions to WS became more aligned between the cultivars at the last sampling point analyzed, and highlighting genotype-dependent behavior of gene expression at the onset of stress. Furthermore, we found that in both cultivars prolonged stress caused more gene induction than gene suppression. By applying a data set exploration approach we retrieved 298 switch genes, mostly representing stress-related processes, that could include candidate master regulators of gene expression of WS status [33]. These switch genes are mostly down regulated in WS, evidencing a negative relation with the majority of genes differentially expressed in WS and suggesting that their down regulation in WS could trigger the activation of drought-related genes. Intriguingly, in the transcriptome analysis of the organ transition from vegetative to mature phase in grapevine, switch genes were up-regulated while the majority of genes involved in grapevine development were down regulated [33]. Taken together these results support a negative regulatory role of the switch genes during a shift to a stressed status or in a developmental phase-transition.

Finally, we investigated the impact of re-watering (RWS) on the physiology and transcriptome of leaves from each cultivar. The RWS in grapevine is a complex process and involves many and different physiological responses [23, 58, 59]. Here, we focused our analysis on the transcriptional differences of the two cultivars upon RWS. The majority of the genes that were modulated during WS reversed their expression profile during the recovery of Montepulciano vines but there was no comparable trend in the Sangiovese cultivar. This suggests that the isohydric Montepulciano leaves undergo such severe stress during WS that most of the genes activated during the response to WS strongly reverse their expression trends, whereas the anisohydric Sangiovese leaves experience less stress and there is no need to counteract the stress response during recovery. Hence, we proposes a novel cause-effect link between the physiological grapevine plant condition (severe vs mild stress) and the intensity of gene expression changes.

## Conclusions

Our transcriptomic comparison of established anisohydric and isohydric cultivars revealed relevant genotype-specific responses (Fig. 8), casting new light on the genetic basis of the proposed classification between isohydric and anisohydric genotypes. Moreover, as drought stress has a strong impact on yield and berry quality, our findings on WS tolerance and plant–water relations obtained by integrating physiology with genomics could be exploited to improve productivity and environmental sustainability of viticulture.

## Methods

### Experimental conditions and layout

This study was conducted in 2011 on 8-year-old potted (60-L) vines of cv. Sangiovese (clone VCR30) and cv. Montepulciano (clone R7) grafted onto 1103 Paulsen rootstock in an outdoor area (Region of Umbria, central Italy, 42°58′ N, 12°24′ E, altitude 405 m above sea level). All the pots were filled with loam soil with a field capacity of 30.2 % [(vol water/vol soil) × 100] and a wilting point of 16.7 %. Each year at the end of February, each vine was pruned to retain four spurs with two buds each. All shoots were oriented upright using suitable stakes. Ten vines per cultivar were used and maintained at about 90 % of maximum water availability (WW, well-watered vines) and ten vines received, from fruit-set to veraison, 40 % of maximum water availability (WS, water-stressed vines) (Fig. 1). During water limitation, all stressed vines were covered with a plastic film to avoid interference due to rainfall and soil water evaporation. The plants were re-watered 46 days after the onset of WS and the post-recovery plant material (RWS) was collected after 24 days (i.e. 70 days after the onset of WS). The water supply per pot was determined by monitoring the soil water content with a Diviner 2000® capacitance probe (Sentek Environ. Tech., Australia) through access tubes located in the pots. In each pot, in June, July and August, the water was supplied every day at 20.00. Throughout the growing season, air temperature and rainfall were monitored by an automatic meteorological station located near the vines.

### Leaf physiological parameters

All parameters were measured 2, 6 and 27 days after achieving 40 % of pot water capacity and 24 days after re-watering. For each treatment and date, leaves between primary shoot nodes 12 and 16 were measured at mid-morning, between 10.00 and 11.00. The leaf area was measured using a leaf area meter (LI-COR Portable Area Meter model LI-3000; LI-COR Environmental, Lincoln, NE). The leaf water potential (Ψ_l_) was measured in 10 leaves (one per vine) using a portable Scholander type pressure chamber (Model 1000, PMS Instruments, Co., USA). Gas exchange readings were taken from 20 individual leaves using a portable LCA-3 infrared gas analyzer (ADC Bio Scientific Ltd, Herts, UK) with air flow adjusted to 350 mL min^−1^. Leaves were sampled under saturating light (PAR >1400 μmol photons m^−2^ s^−1^) and the photosynthetic rate (A_max_) and stomatal conductance (g_s_) were calculated from inlet and outlet CO_2_ and H_2_O concentrations. Intrinsic water use efficiency was then determined using the equation WUE_i_ = A_max_/g_s_. Fluorescence transients (F_v_/F_m_ ratio) were measured on the same leaves using a Handy-Pea fluorimeter (Hansatech Institute Ltd, Norfolk, UK). Dark adaptation was achieved by covering the analyzed area with a leaf clip for at least 20 min, opening the shutter and exposing the dark-adapted leaf tissue to an actinic light flash (650 nm, intensity >3000 μmol photons m^−2^ s^-1^). F_v_ (variable fluorescence) was calculated as the difference between F_m_ and F_o_, where F_o_ is the ground fluorescence [60]. The area above the fluorescence curve between F_o_ and F_m_ (*Area*), which indicates the plastoquinone (Q_a_) pool size on the reducing size of PSII, was also calculated automatically.

### Leaf biochemical parameters

The same leaves described above were also used for the analysis of chlorophyll and carotenoids. Three samples per treatment, each consisting of eight pieces of different leaves taken between primary shoot nodes 12 and 16, were frozen in liquid N_2_ and stored a −80 °C. Pigments were extracted under subdued light to avoid the degradation or isomerization of carotenoids [61]. We used 2 μg of *trans*-β-apo-8′-carotenal as an internal standard. Samples were separated by reversed-phase high-performance liquid chromatography (RP-HPLC) using an Agilent 1260HPLC system (Agilent Technologies, Palo Alto, California, USA) equipped with a diode array detector (DAD) and a YMC30 column (3 μm, 150 × 4.6 mm internal diameter) protected by a guard column (5 μm, 10 × 4 mm internal diameter) both from YMC (Europe, Schermbeck, Germany). G1315C Agilent Chem Station software was used for data processing. Pigments were identified by comparing retention times and spectral properties to the following authentic standards: *trans*-β-carotene, chlorophyll *a* and *b*, *trans*-β-apo-8′-carotenal and lutein (Sigma-Aldrich, St. Louis, MO, USA), lutein epoxide, zeaxanthin, neoxanthin, violaxanthin and antheraxanthin (Carote Nature GmbH, Lupsingen, Switzerland) prepared from 1 mg mL^−1^ stocks in chloroform containing 0.1 % butylated hydroxytoluene (BHT).

Catalase (CAT) specific activity (presented as μmol H_2_O_2_ consumed by the enzyme during 1 min of linearity per g of fresh tissue and/or per mg of total protein) was determined as described by Ozden et al. [62].

Foliar ABA was extracted following the procedure described by Vilarò et al. [63] with some modifications. The frozen leaf material was weighted (fresh weight) and lyophilised (LIO5P, 5Pascal, Trezzano, Italy). Lyophilised material was weighted (dry weight) and ground (MF10, IKAlabortechnik, Staufen, Germany). Leaf material (0.1 g) was extracted with 10 ml of methanol/water (1:1 v/v, pH = 3 with formic acid) for 30 min using a ultrasonic bath. After centrifugation, the supernatant was filtered through a paper filter and the same procedure was repeated for the remaining pellet. The collected filtrates were extracted twice with dichloromethane (15 ml) and the organic phase evaporated under vacuum. The residue was dissolved to a 1 ml with acetone and water/acetonitrile (50:50 *v/v*, 0.1 % formic acid) for the HPLC analysis. Analytical standards of (±) Abscisic acid (purity ≥98.5 %) was purchased from Sigma-Aldrich, PA-grade methanol, acetone, dichloromethane and formic acid, and HPLC-grade acetonitrile and water were purchased from VWR Chemicals. Analyses were performed on a Perkin-Elmer PE 200 system (Autosampler, Binary Pump and UV-VIS detector) equipped with an IB-Sil C8-HC (5 mm × 250 mm × 4.6 mm Phenomenex) column and IB-Sil C8 (5 mm × 30 mm × 4.6 mm Phenomenex precolumn at a flow rate of 0.8 mL min^−1^; the injection volume was 20 μL and the detection was made at 270 nm. The mobile phase of acetonitrile/water (30:70 *v/v*, 0.1 % formic acid) was previously filtered and degassed. The compound was identified by comparing the retention times with those of authentic reference compound. The peaks were quantified by an external standard method, using the measurements of the peak areas and a calibration curve. Stock solutions of ABA standards were prepared by diluting a solution (10 mg ml^−1^ in acetonitrile) to obtain a range of concentrations from 0.01 to 10 mg ml^−1^. The limit of detection (LOD) was 0.005 mg l^−1^.

The H_2_O_2_ concentration (presented as μmol H_2_O_2_ per g of fresh tissue and/or per mg of total protein) was determined essentially as described by Loreto and Velikova [64] and calculated from a standard curve plotted in the range 100–1000 μmol/ml. For both assays, readings were recorded on a Lambda 3B UV-vis spectrophotometer (Perkin-Elmer Instruments Ltd, Seer Green, Beaconsfield, UK).

### Statistical analysis

All physiological data were processed by two-way analysis of variance (ANOVA; genotype vs. water treatment) using SigmaStat software (SPSS Science, USA). Treatments were compared using the Student-Newman-Keuls test (*p* ≤ 0.05).

### Microarray analysis

As stated above, leaf samples were taken after 2, 6 and 27 days of WS, and 24 days after re-watering (three pools of leaves, each consisting of four pieces). At the same times, three pools of 15 berries were taken from the bunch opposite the node corresponding to the leaf sample. All samples were immediately frozen in liquid N_2_ and stored at −80 °C.

Total RNA was extracted from ~50 mg of frozen leaves and ~200 mg of berry (pericarp plus seeds) using the Spectrum™ Plant Total RNA kit (Sigma-Aldrich) as previously described [65]. We hybridized 5 μg of total RNA per sample to a NimbleGen microarray 090818_Vitus_exp_HX12 chip (Roche, NimbleGen Inc., Madison, WI), according to the manufacturer’s instructions [65]. Statistical analysis of the microarray data was carried out using TMeV v4.8 (mev.tm4.org/). Statistical analysis of microarrays (SAM) was carried out with a false discovery rate (FDR) of 0.01 % and ANOVA was carried out using α = 0.01 and standard Bonferroni correction. Hierarchical cluster analysis was applied using Pearson’s correlation distance, unless stated otherwise. Principal component analysis (PCA) was carried out using SIMCA P+ v13 (Umetrics, USA). Gene Ontology (GO) annotation was applied to gene clusters and organ-specific genes using the BiNGO v2.3 plug-in tool in Cytoscape v2.6 with PlantGOslim categories, as described by Maere et al. [66]. Overrepresented PlantGOslim categories were identified using a hypergeometric test with a significance threshold of 0.01 for genes modulated in leaves and 0.05 for genes modulated in berries. STEM v1.3.8 was used for clustering, comparing and visualizing gene expression data [26]. Line plots were drawn using SigmaPlot v13.0.

## Additional files

Additional file 1: Changes in physiological and biochemical parameters of Sangiovese (SG) and Montepulciano (MP) vines under well-watered control (WW) and water stress (WS) conditions. Data were taken 2, 6 and 27 days after WS. For each measurement date, the means ± SE followed by different letters are significantly different at *p* < 0.05 according to the Student-Newman-Keuls test. (DOC 106 kb)
Additional file 2: The 5947 genes significantly modulated in leaves under our experimental conditions. (XLSX 2645 kb)
Additional file 3: The 1034 annotated genes significantly modulated in leaves with a FC value >2 in at least one cultivar under WS. The data set indicates whether a gene was retrieved by the 12-class approach (12C), by the 6-class approach (6C) or if it was shared (S) between the two approaches. (XLSX 160 kb)
Additional file 4: STEM analysis of the 1034 genes modulated in leaves. (XLSX 90 kb)
Additional file 5: List of the differentially modulated genes clustered by time points (T1, T2 and T3). (XLSX 251 kb)
Additional file 6: List of the 1236 genes differentially modulated between WS and WW conditions regardless of the genotype. (XLSX 198 kb)
Additional file 7: Coexpression network. (TIF 262 kb)
Additional file 8: List of genes differentially modulated in leaves during recovery from water stress (RWS). (XLSX 318 kb)
Additional file 9: List of the 11,389 genes significantly modulated in berries under our experimental conditions. (XLSX 4896 kb)
Additional file 10: List of the 269 annotated genes significantly modulated in berries with a FC value >2 in at least one cultivar under WS. (XLSX 48 kb)
Additional file 11: STEM analysis on the 269 modulated genes in berries. (XLSX 959 kb)

## Abbreviations

ABA: Abscisic acid
ANOVA: Analysis of variance
HSP: Heat shock proteins
PCA: Principal component analysis
ROS: Reactive oxygen species
RWS: Revived water stress
SAM: Significant analysis of microarray
WS: Water stressed
WW: Well watered

## References

[1] Vivier MA, Pretorius IS (2002). Genetically tailored grapevines for the wine industry. Trends Biotechnol.

[2] Hannah L, Roehrdanz PR, Ikegami M, Shepard AV, Shaw MR, Tabor G, Zhi L, Marquet PA, Hijmans RJ (2013). Climate change, wine, and conservation. Proc Natl Acad Sci U S A.

[3] Alleweldt G, Dettweiler-Munch E (1992). The genetic resources of Vitis. Genetic and geographic origin of grape cultivars, their prime names and synonyms.

[4] Chaves MM, Zarrouk O, Francisco R, Costa JM, Santos T, Regalado AP, Rodrigues ML, Lopes CM (2010). Grapevine under deficit irrigation: hints from physiological and molecular data. Ann Bot.

[5] Smart RE, Coombe BG, Kozlowski TT (1983). Water relations of grapevines. Additional woody crop plants. Water Deficiencies and Plant Growth.

[6] Jones HD, Turner NC, Kramer PJ (1980). Interaction and integration of adaptive responses to water stress: the implication of an unpredictable environment. Adaptation of Plants to Water and High Temperature Stress.

[7] Stocker O, Ruhland W (1956). Die abhängigkeit der transpiration von den umweltfaktoren. Encyclopedia of Plant Physiology.

[8] Tardieu F, Simonneau T (1998). Variability among species of stomatal control under fluctuating soil water status and evaporative demand: modelling isohydric and anisohydric behaviours. J Exp Bot.

[9] Schultz HR (2003). Differences in hydraulic architecture account for near-isohydric and anisohydric behaviour of two field-grown Vitis vinifera L. cultivars during drought. Plant Cell Environ.

[10] Chaves MM, Oliveira MM (2004). Mechanisms underlying plant resilience to water deficits: prospects for water-saving agriculture. J Exp Bot.

[11] Merli MC, Gatti M, Galbignani M, Bernizzoni F, Magnanini E, Poni S (2015). Water use efficiency in Sangiovese grapes (Vitis vinifera L.) subjected to water stress before veraison: different levels of assessment lead to different conclusions. Funct Plant Biol.

[12] Poni S, Bernizzoni F, Civardi S, Gatti M, Porro D, Camin F (2009). Performance and water-use efficiency (single-leaf vs. whole-canopy) of well-watered and half-stressed split-root Lambrusco grapevines grown in Po Valley (Italy). Agric Ecosyst Environ.

[13] Palliotti A, Tombesi S, Frioni T, Famiani F, Silvestroni O, Zamboni M, Poni S (2014). Morpho-structural and physiological response of container-grown Sangiovese and Montepulciano cvv. (Vitis vinifera) to re-watering after a pre-veraison limiting water deficit. Funct Plant Biol.

[14] Castellarin SD, Matthews MA, Di Gaspero G, Gambetta GA (2007). Water deficits accelerate ripening and induce changes in gene expression regulating flavonoid biosynthesis in grape berries. Planta.

[15] Deluc LG, Quilici DR, Decendit A, Grimplet J, Wheatley MD, Schlauch KA, Merillon JM, Cushman JC, Cramer GR (2009). Water deficit alters differentially metabolic pathways affecting important flavor and quality traits in grape berries of Cabernet Sauvignon and Chardonnay. BMC Genomics.

[16] Daszkowska-Golec A, Szarejko I (2013). Open or close the gate - stomata action under the control of phytohormones in drought stress conditions. Front Plant Sci.

[17] Kim TH, Bohmer M, Hu HH, Nishimura N, Schroeder JI (2010). Guard Cell Signal Transduction Network: Advances in Understanding Abscisic Acid, CO2, and Ca2+ Signaling. Annu Rev Plant Biol.

[18] Huang DQ, Wu WR, Abrams SR, Cutler AJ (2008). The relationship of drought-related gene expression in Arabidopsis thaliana to hormonal and environmental factors. J Exp Bot.

[19] Nemhauser JL, Hong FX, Chory J (2006). Different plant hormones regulate similar processes through largely nonoverlapping transcriptional responses. Cell.

[20] Berdeja M, Nicolas P, Kappel C, Dai ZW, Hilbert G, Peccoux A, Lafontaine M, Ollat N, Gomès E, Delrot S (2015). Water limitation and rootstock genotype interact to alter grape berry metabolism through transcriptome reprogramming. Hortic Res.

[21] Corso M, Vannozzi A, Maza E, Vitulo N, Meggio F, Pitacco A, Telatin A, D’Angelo M, Feltrin E, Negri AS (2015). Comprehensive transcript profiling of two grapevine rootstock genotypes contrasting in drought susceptibility links the phenylpropanoid pathway to enhanced tolerance. J Exp Bot.

[22] Cramer GR, Ergul A, Grimplet J, Tillett RL, Tattersall EAR, Bohlman MC, Vincent D, Sonderegger J, Evans J, Osborne C (2007). Water and salinity stress in grapevines: early and late changes in transcript and metabolite profiles. Funct Integr Genomics.

[23] Flexas J, Baron M, Bota J, Ducruet JM, Galle A, Galmes J, Jimenez M, Pou A, Ribas-Carbo M, Sajnani C (2009). Photosynthesis limitations during water stress acclimation and recovery in the drought-adapted Vitis hybrid Richter-110 (V-berlandierixV-rupestris). J Exp Bot.

[24] Perrone I, Pagliarani C, Lovisolo C, Chitarra W, Roman F, Schubert A (2012). Recovery from water stress affects grape leaf petiole transcriptome. Planta.

[25] Tombesi S, Nardini A, Farinelli D, Palliotti A (2014). Relationships between stomatal behavior, xylem vulnerability to cavitation and leaf water relations in two cultivars of Vitis vinifera. Physiol Plant.

[26] Ernst J, Nau GJ, Bar-Joseph Z (2005). Clustering short time series gene expression data. Bioinformatics.

[27] Liu JY, Chen NN, Chen F, Cai B, Dal Santo S, Tornielli GB, Pezzotti M, Cheng ZMM (2014). Genome-wide analysis and expression profile of the bZIP transcription factor gene family in grapevine (Vitis vinifera). BMC Genomics.

[28] Koike M, Takezawa D, Arakawa K, Yoshida S (1997). Accumulation of 19-kDa plasma membrane polypeptide during induction of freezing tolerance in wheat suspension-cultured cells by abscisic acid. Plant Cell Physiol.

[29] Hashimoto M, Negi J, Young J, Israelsson M, Schroeder JI, Iba K (2006). Arabidopsis HT1 kinase controls stomatal movements in response to CO2. Nat Cell Biol.

[30] Suzuki N, Mittler R (2006). Reactive oxygen species and temperature stresses: A delicate balance between signaling and destruction. Physiol Plant.

[31] Gill SS, Tuteja N (2010). Reactive oxygen species and antioxidant machinery in abiotic stress tolerance in crop plants. Plant Physiol Biochem.

[32] Verslues PE, Agarwal M, Katiyar-Agarwal S, Zhu JH, Zhu JK (2006). Methods and concepts in quantifying resistance to drought, salt and freezing, abiotic stresses that affect plant water status. Plant J.

[33] Palumbo MC, Zenoni S, Fasoli M, Massonnet M, Farina L, Castiglione F, Pezzotti M, Paci P (2014). Integrated Network Analysis Identifies Fight-Club Nodes as a Class of Hubs Encompassing Key Putative Switch Genes That Induce Major Transcriptome Reprogramming during Grapevine Development. Plant Cell.

[34] Al-Quraan NA, Locy RD, Singh NK (2010). Expression of calmodulin genes in wild type and calmodulin mutants of Arabidopsis thaliana under heat stress. Plant Physiol Biochem.

[35] Andrade MA, Bork P (1995). Heat Repeats in the Huntingtons-Disease Protein. Nat Genet.

[36] Collart MA, Panasenko OO (2012). The Ccr4-Not complex. Gene.

[37] Hershko A, Ciechanover A (1998). The ubiquitin system. Annu Rev Biochem.

[38] Lorenz DH, Eichhorn KW, Bleiholder H, Klose R, Meier U, Weber E (1994). Phänologische Entwicklungsstadien der Rebe (Vitis vinifera L. ssp. vinifera). Codierung und Beschreibung nach der erweiterten BBCH-Skala. Vitic Enol Sci.

[39] Young PR, Lashbrooke JG, Alexandersson E, Jacobson D, Moser C, Velasco R, Vivier MA (2012). The genes and enzymes of the carotenoid metabolic pathway in Vitis vinifera L. BMC Genomics.

[40] Tramontini S, Vitali M, Centioni L, Schubert A, Lovisolo C (2013). Rootstock control of scion response to water stress in grapevine. Environ Exp Bot.

[41] Hubbard KE, Nishimura N, Hitomi K, Getzoff ED, Schroeder JI (2010). Early abscisic acid signal transduction mechanisms: newly discovered components and newly emerging questions. Genes Dev.

[42] Hochberg U, Degu A, Fait A, Rachmilevitch S (2013). Near isohydric grapevine cultivar displays higher photosynthetic efficiency and photorespiration rates under drought stress as compared with near anisohydric grapevine cultivar. Physiol Plant.

[43] Nambara E, Marion-Poll A (2005). Abscisic acid biosynthesis and catabolism. Annu Rev Plant Biol.

[44] Brodribb TJ, Holbrook NM (2003). Stomatal closure during leaf dehydration, correlation with other leaf physiological traits. Plant Physiol.

[45] Schymanski SJ, Or D, Zwieniecki M (2013). Stomatal Control and Leaf Thermal and Hydraulic Capacitances under Rapid Environmental Fluctuations. PloS One.

[46] Mittler R (2006). Abiotic stress, the field environment and stress combination. Trends Plant Sci.

[47] Palliotti A, Tombesi S, Frioni T, Silvestroni O, Lanari O, D’Onofrio C, Matarese F, Bellincontro A, Poni S (2015). Physiological parameters and protective energy dissipation mechanisms expressed in the leaves of two Vitis vinifera L. genotype under multiple summer stresses. J Plant Physiol.

[48] Feder ME, Hofmann GE (1999). Heat-shock proteins, molecular chaperones, and the stress response: Evolutionary and Ecological Physiology. Annu Rev Physiol.

[49] Wang WX, Vinocur B, Altman A (2003). Plant responses to drought, salinity and extreme temperatures: towards genetic engineering for stress tolerance. Planta.

[50] Krasensky J, Jonak C (2012). Drought, salt, and temperature stress-induced metabolic rearrangements and regulatory networks. J Exp Bot.

[51] Paul MJ, Primavesi LF, Jhurreea D, Zhang YH (2008). Trehalose metabolism and signaling. Annu Rev Plant Biol.

[52] Avonce N, Leyman B, Mascorro-Gallardo JO, Van Dijck P, Thevelein JM, Iturriaga G (2004). The Arabidopsis trehalose-6-P synthase AtTPS1 gene is a regulator of glucose, abscisic acid, and stress signaling. Plant Physiol.

[53] Ge LF, Chao DY, Shi M, Zhu MZ, Gao JP, Lin HX (2008). Overexpression of the trehalose-6-phosphate phosphatase gene OsTPP1 confers stress tolerance in rice and results in the activation of stress responsive genes. Planta.

[54] Li HW, Zang BS, Deng XW, Wang XP (2011). Overexpression of the trehalose-6-phosphate synthase gene OsTPS1 enhances abiotic stress tolerance in rice. Planta.

[55] Lunn JE, Delorge I, Figueroa CM, Van Dijck P, Stitt M (2014). Trehalose metabolism in plants. Plant J.

[56] Kaplan F, Guy CL (2004). Beta-amylase induction and the protective role of maltose during temperature shock. Plant Physiol.

[57] Taji T, Ohsumi C, Iuchi S, Seki M, Kasuga M, Kobayashi M, Yamaguchi-Shinozaki K, Shinozaki K (2002). Important roles of drought- and cold-inducible genes for galactinol synthase in stress tolerance in Arabidopsis thaliana. Plant J.

[58] Lovisolo C, Perrone I, Hartung W, Schubert A (2008). An abscisic acid-related reduced transpiration promotes gradual embolism repair when grapevines are rehydrated after drought. New Phytol.

[59] Zufferey V, Cochard H, Ameglio T, Spring JL, Viret O (2011). Diurnal cycles of embolism formation and repair in petioles of grapevine (Vitis vinifera cv. Chasselas). J Exp Bot.

[60] Strasser RJ, Srivastava A, Govindjee (1995). Polyphasic Chlorophyll-Alpha Fluorescence Transient in Plants and Cyanobacteria. Photochem Photobiol.

[61] Lashbrooke JG, Young PR, Strever AE, Stander C, Vivier MA (2010). The development of a method for the extraction of carotenoids and chlorophylls from grapevine leaves and berries for HPLC profiling. Aust J Grape Wine Res.

[62] Ozden M, Demirel U, Kahraman A (2009). Effects of proline on antioxidant system in leaves of grapevine (Vitis vinifera L.) exposed to oxidative stress by H2O2. Sci Hortic.

[63] Vilaro F, Canela-Xandri A, Canela R (2006). Quantification of abscisic acid in grapevine leaf (Vitis vinifera) by isotope-dilution liquid chromatography-mass spectrometry. Anal Bioanal Chem.

[64] Loreto F, Velikova V (2001). Isoprene produced by leaves protects the photosynthetic apparatus against ozone damage, quenches ozone products, and reduces lipid peroxidation of cellular membranes. Plant Physiol.

[65] Fasoli M, Dal Santo S, Zenoni S, Tornielli GB, Farina L, Zamboni A, Porceddu A, Venturini L, Bicego M, Murino V (2012). The Grapevine Expression Atlas Reveals a Deep Transcriptome Shift Driving the Entire Plant into a Maturation Program. Plant Cell.

[66] Maere S, Heymans K, Kuiper M (2005). BiNGO: a Cytoscape plugin to assess overrepresentation of Gene Ontology categories in Biological Networks. Bioinformatics.

