# Supplementary material for: Distinct transcriptome responses to water limitation in isohydric and anisohydric grapevine cultivars
Source: BMC Genomics. 2016 Oct 20;17:815. doi: 10.1186/s12864-016-3136-x (PMC5073746; doi:10.1186/s12864-016-3136-x)
Supplement: Additional file 1: — Changes in physiological and biochemical parameters of Sangiovese (SG) and Montepulciano (MP) vines under well-watered control (WW) and water stress (WS) conditions. Data were taken 2, 6 and 27 days after WS. For each measurement date, the means ± SE followed by different letters are significantly different at p < 0.05 according to the Student-Newman-Keuls test. (DOC 106 kb) [file 12864_2016_3136_MOESM1_ESM.doc]

**Supporting Information File S1**

**Table 1.** Changes in chlorophylls and carotenoids content (expressed as μg/g DW) and catalase (CAT) activity and hydrogen peroxide (H_2_O_2_) in leaves of Sangiovese and Montepulciano grapevines subjected to early deficit irrigation (WS) and well watered (WW). Data were taken 2 days after water deprivation during 10.00-11.00 hr interval. Mean values within rows were separated by Student-Newman-Keuls (SNK) test where different letter indicate statistical outcomes.

| Cultivar 🡺 | Sangiovese | |  | Montepulciano | |
| --- | --- | --- | --- | --- | --- |
| Water regime 🡺 | WW | WS |  | WW | WS |
| Chl *a* | 6198 b | 8079 a |  | 8449 a | 7896 a |
| Chl *b* | 3440 b | 4021 a |  | 4057 a | 3691 ab |
| Chl *a*/*b* | 1.82 c | 2.01 b |  | 2.09 ab | 2.14 a |
| Violaxanthin | 370.7 | 411.0 |  | 396.9 | 416.5 |
| Antheraxanthin | 63.5 | 65.5 |  | 63.2 | 70.6 |
| Zeaxanthin | 77.3 | 77.2 |  | 87.9 | 95.6 |
| V+A+Z | 511.5 | 553.6 |  | 548.0 | 582.7 |
| DEPS* | 0.21 | 0.20 |  | 0.22 | 0.22 |
| Neoxanthin | 574.5 | 655.9 |  | 665.6 | 608.5 |
| Lutein | 628.9 | 746.0 |  | 739.2 | 700.0 |
| β-carotene | 1179 | 1309 |  | 1301 | 1206 |
| Car_total_ | 2894 | 3264 |  | 3256 | 3098 |
| Car_total_/Chl_total_  | 0.29 | 0.27 |  | 0.26 | 0.27 |
|  |  |  |  |  |  |
| H_2_O_2_ (μmol/mg protein) | 21.0 a | 18.0 a |  | 14.5 b | 22.0 a |
| CAT activity (μmol H_2_O_2_ min^-1^ mg^-1^ protein) | 8.0 a | 7.1 ab |  | 5.1 b | 8.6 a |

*DEPS (de-epoxidation state) = [(Z + 0.5 A)/(V + A + Z)]

**Table 2.** Changes in chlorophylls and carotenoids content (expressed as μg/g DW) and catalase (CAT) activity and hydrogen peroxide (H_2_O_2_) in leaves of Sangiovese and Montepulciano grapevines subjected to early deficit irrigation (WS) and well watered (WW). Data were taken 6 days after water deprivation during 10.00-11.00 hr interval. Mean values within rows were separated by Student-Newman-Keuls (SNK) test where different letter indicate statistical outcomes

| Cultivar 🡺 | Sangiovese | |  | Montepulciano | |
| --- | --- | --- | --- | --- | --- |
| Water regime 🡺 | WW | WS |  | WW | WS |
| Chl *a* | 6128 b | 7939 a |  | 7897 a | 8245 a |
| Chl *b* | 3213 b | 3568 a |  | 3897 a | 3798 a |
| Chl *a*/*b* | 1.91 b | 2.22 a |  | 2.04 ab | 2.17 a |
| Violaxanthin | 279.3 b | 339.1 a |  | 310.5 a | 277.0 b |
| Antheraxanthin | 51.5 b | 72.4 a |  | 62.5 ab | 74.6 a |
| Zeaxanthin | 97.2 b | 120.2 b |  | 116.1 b | 170.6 a |
| V+A+Z | 428.0 b | 531.7 a |  | 489.9 a | 522.2 a |
| DEPS* | 0.29 b | 0.29 b |  | 0.30 b | 0.40 a |
| Neoxanthin | 521.6 b | 559.3 ab |  | 600.2 a | 604.3 a |
| Lutein | 601.5 b | 760.0 a |  | 705.2 a | 781.3 a |
| β-carotene | 1070 c | 1498 a |  | 1213 b | 1352 a |
| Car_total_ | 2622 b | 3350 a |  | 3001 a | 3257 a |
| Car_total_/Chl_total_ | 0.28 | 0.29 |  | 0.26 | 0.27 |
|  |  |  |  |  |  |
| H_2_O_2_ (μmol/mg protein) | 17.7 a | 16.4 a |  | 11.3 b | 15.8 a |
| CAT activity (μmol H_2_O_2_ min^-1^ mg^-1^ protein) | 3.2 ab | 2.2 b |  | 2.1 b | 3.9 a |

*DEPS (de-epoxidation state) = [(Z + 0.5 A)/(V + A + Z)]

**Table 3.** Changes in chlorophylls and carotenoids content (expressed as μg/g DW) and catalase (CAT) activity and hydrogen peroxide (H_2_O_2_) in leaves of Sangiovese and Montepulciano grapevines subjected to early deficit irrigation (WS) and well watered (WW). Data were taken 27 days after water deprivation during 10.00-11.00 hr interval. Mean values within rows were separated by Student-Newman-Keuls (SNK) test where different letter indicate statistical outcomes

| Cultivar 🡺 | Sangiovese | |  | Montepulciano | |
| --- | --- | --- | --- | --- | --- |
| Water regime 🡺 | WW | WS |  | WW | WS |
| Clorophylls and carotenoids (μg/g DW) | | | | | |
| Chl *a* | 5213 b | 7653 a |  | 6912 a | 6942 a |
| Chl *b* | 2551 b | 3616 a |  | 3345 a | 3260 a |
| Chl *a*/*b* | 2.04 | 2.12 |  | 2.07 | 2.13 |
| Violaxanthin | 206.1 b | 216.2 b |  | 303.8 a | 249.3 b |
| Antheraxanthin | 57.3 c | 112.8 a |  | 42.7 c | 73.7 b |
| Zeaxanthin | 56.4 c | 228.6 a |  | 38.6 c | 136.7 b |
| V+A+Z | 319.8 c | 557.6 a |  | 395.1 b | 459.7 b |
| DEPS* | 0.27 b | 0.51 a |  | 0.16 c | 0.36 b |
| Neoxanthin | 386.2 b | 548.5 a |  | 529.8 a | 512.0 a |
| Lutein | 498.2 b | 690.9 a |  | 641.2 a | 641.0 a |
| β-carotene | 873.1 c | 1341 a |  | 989.1 b | 1071 b |
| Car_total_ | 2076 c | 3138 a |  | 2646 b | 2594 b |
| Car_total_/Chl_total_ | 0.27 | 0.28 |  | 0.26 | 0.26 |
|  |  |  |  |  |  |
| H_2_O_2_ (μmol/mg protein) | 26.3 a | 24.9 a |  | 15.7 c | 21.3 b |
| CAT activity (μmol H_2_O_2_ min^-1^ mg^-1^ protein) | 4.7 c | 3.5 c |  | 8.1 b | 12.7 a |

*DEPS (de-epoxidation state) = [(Z + 0.5 A)/(V + A + Z)]

**Table 4.** Vegetative growth and canopy characteristics in Sangiovese and Montepulciano (*Vitis vinifera*) grapevines subjected to well-watered conditions (WW) and re-watered after a pre-veraison deficit irrigation (WS).

Significant interactions are indicated: *, P < 0.05; **, P < 0.01; ***, P < 0.001; ns (not significant), P > 0.05 respectively. Mean values were pooled over years and separated, within each row with different letters, by using Student–Newman–Keuls test

| Parameter | Sangiovese | | | | Montepulciano | | | | Significance of genotype × water deficit interaction | |
| --- | --- | --- | --- | --- | --- | --- | --- | --- | --- | --- |
|  | WW | | WS | | WW | | WS | |  | |
| Shoots per vine | 7.3 | 7.4 | | 7.1 | | 7.5 | | ns | |  |
| Primary shoot length (cm) | 215 a | 148 c | | 173 b | | 115 d | | *** | |  |
| Internode length (cm) | 7.9 a | 6.5 b | | 6.0 b | | 4.6 c | | * | |  |
| Internode diameter (3°-4° nodes) (mm) | 8.7 b | 7.5 c | | 9.9 a | | 8.8 b | | ** | |  |
| Lateral shoots (n°/vine) | 42.3 b | 22.3 c | | 62.1 a | | 19.3 c | | ** | |  |
| Lateral shoot length (cm) | 35.6 a | 19.6 c | | 25.9 b | | 14.2 d | | *** | |  |
| Leaf area from primary shoots (m^2^ vine^-1^)^A^ | 2.80 a | 1.15 d | | 2.49 b | | 1.62 c | | *** | |  |
| Leaf area from lateral shoots (m^2^ vine^-1^) | 1.23 b | 0.62 c | | 1.58 a | | 0.49 d | | ** | |  |
| Pruning weight (g vine^-1^) | 0.38 a | 0.27 b | | 0.39 a | | 0.21 c | | ** | |  |

^A^In Sangiovese vines, about 0.38 m^2^ leaf area per vine (basal leaves) showed early yellowing and drop
