# Supplementary figures and images for: Distinct transcriptome responses to water limitation in isohydric and anisohydric grapevine cultivars
Source: BMC Genomics. 2016 Oct 20;17:815. doi: 10.1186/s12864-016-3136-x (PMC5073746; doi:10.1186/s12864-016-3136-x)

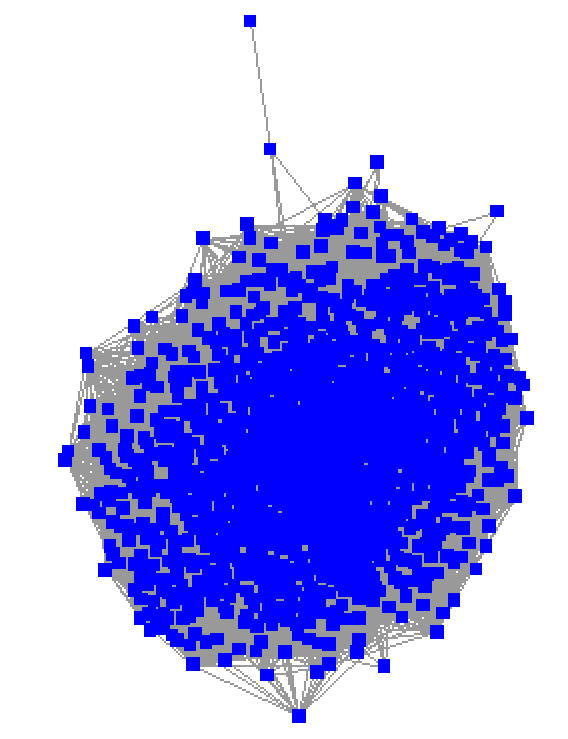

Supplement: Additional file 7: — Coexpression network. (TIF 262 kb) [file 12864_2016_3136_MOESM7_ESM.tif]
